# The association between workloads and health hazard among delivery riders in China

**DOI:** 10.3389/fpubh.2025.1603087

**Published:** 2025-05-30

**Authors:** Ai-Lin Mao, Chun-Miao Tian, Chen-Xi Liu

**Affiliations:** School of Labor Economics, Capital University of Economics and Business, Beijing, China

**Keywords:** delivery riders, occupational health, job demands, risk management, gig workers

## Abstract

**Introduction:**

Drawing on the extended job demands-resources model, this study aims to identify the primary occupational health risks affecting delivery riders and explore mechanisms between workload and illness.

**Methods:**

A respondent-driven sampling method was employed to minimize data bias. A total of 1,092 riders in Beijing, Shanghai, and Jinan participated in the survey. Logit regression analysis was conducted to assess the associations and mechanisms were also analyzed.

**Results:**

A significant positive relationship was observed between the number of daily deliveries and daily working hours with reported illness. This association was more significant among riders who are primary family breadwinners or who work part-time.

**Conclusion:**

Excessive workload negatively affects the health of delivery riders. Overwork may heighten riders’ risk perception, which can ultimately lead to illness. However, this relationship can be mitigated if delivery platforms implementing measures to reduce work-related pressure. A key practical implication of this study is the urgent need for platform companies to assume greater responsibility in labor protection, particularly in curbing the tendency toward overwork.

## 1 Introduction

Online delivery services have rapidly expanded due to the growth of the platform economy and advancements in technology (1). As a central component of the services, delivery riders (DRs) benefit from the flexibility inherent in gig work. Yet they also face significant risks arising from its uncertain and precarious nature (2). A keyword search using “online delivery riders” and “traffic accidents” on China Judgments Online (a website that publishes all legally effective rulings, except those with special legal provisions) yielded 690 rulings related to DRs’ traffic accidents between 2020 and 2023 (3).

While injury prevention remains a pressing issue in the labor protection of DRs, precarious employment has also been linked to a range of adverse health outcomes (4). Some researchers have addressed this issue from two key aspects. First, the current occupational health and safety legal framework is based on conventional employer-employee binary, which makes it challenging to integrate gig workers into the occupational disease prevention and control system (5). Second, the lack of social insurance amplifies the consequences of health impairments among gig workers (6). Furthermore, platforms often neglect to provide occupational safety training and to establish appropriate management systems in pursuit of cost reductions. As a result, workers are continuously exposed to high-intensity, high-risk working environments (7).

As a crucial dimension of occupational safety and health, it is essential to examine the health risk faced by vulnerable groups and identify actionable counter-measures to support the well-being of DR. A handful of studies have investigated the health determinants affecting DR (4, 8), but research in this area is still limited. In particular, the occupational burden and the mechanisms linking gig work to health outcomes remain understudied.

To address this gap, the present study focuses on Chinese DRs as representative gig workers. It applies the Job Demands-Resources (JD-R) model to investigate the relationship between workload and health. The goal is to identify the key health risk factors associated with gig work and provide informed policy recommendations for labor protection, ultimately contributing to this group’s sustainable development and human capital accumulation.

## 2 Background and literature review

### 2.1 Background

According to data released by China’s two largest online delivery platforms, the total number of registered DRs has exceeded 5.7 million (9, 10). Official progressions estimate that the number of Chinese food DRs will surpass 10 million in the near future (11). These DRs broadly categorized into two types: *Zhuansong* DRs and *Zhongbao* DRs (12). *Zhuansong* DRs are formally employed under labor contracts with platform companies and are subject to direct daily management at designated work sites. In contrast, *Zhongbao* DRs voluntarily register with the platforms to provide delivery services. They operate more independently, accepting and fulfilling orders at their discretion (13).

Despite differences in employment status and management, both Zhuansong and Zhongbao DRs follow similar workflows, which generally include three phases: waiting, receiving/picking up, and delivering orders. First, riders must open the platform’s mobile app to log in and go online, entering a waiting phase during which the system assigns them orders. Zhuansong DRs are required to meet a minimum daily online time, while Zhongbao DRs are not subject to such a requirement.

Second, once a consumer places an order, the platform allocates it to a DR based on several factors, including the rider’s proximity to the customer and merchant, current workload, historical rejection rate, and external conditions such as weather and traffic. Zhuansong DRs are not permitted to refuse assigned orders, although they may transfer up to three orders per day to other riders. Zhongbao DRs, on the other hand, can reject assigned orders; however, each rejection is recorded by the system. As the refusal rate increases, the number of future assigned orders decreases. If an order is not accepted promptly, the system issues multiple reminders. Persistent inaction leads to reassignment of the order and a penalty for the DR.

Orders rejected by DRs are returned to a shared “order pool” accessible to all riders. DRs refer to this process of getting orders as “snatching orders.” Both riders waiting for orders and those currently delivering may participate in the “game of snatching,” where speed is the sole determinant of success (43). This competitive process contributes to the reasons foods DRs deliver get involved in accidents. Typically, most orders for Zhuansong DRs are system-assigned, whereas Zhongbao DRs rely more heavily on the snatching mechanism.

Third, the final step involves picking up and delivering the food. Most platforms allocate approximately 30 min for this process, from the time the order is accepted to when it is delivered to the customer. This time frame is often tight, especially considering riders must wait for merchants to prepare the food before pickup. The stereotypical image of a DR is someone speeding through traffic while a mobile phone repeatedly warns: “You are out of time.”

In addition to these standard procedures, DRs often handle multiple orders simultaneously. Their common strategy is to accept orders, align geographically, or even merge deliveries to increase efficiency and maximize income.

After an order is completed, platforms require customers to evaluate the DR’s performance based on service quality and whether the delivery exceeded the expected time. Ratings typically range from “very dissatisfied” to “very satisfied.” Delivery riders receive rewards based on customer evaluations and additional performance indicators. Platforms generally offer two types of rewards: cash and virtual points (14). Cash rewards commonly include attendance bonuses (usually for Zhuansong DRs who work for a minimum number of days per month), delivery volume bonuses (for completing certain orders within a specific timeframe), and subsidies for adverse weather conditions.

Virtual points, on the other hand, are linked to a DR’s “level,” which is directly related to their income. Higher-level DRs receive preferential treatment, such as priority access to high-value orders and higher commission rates. Table 1 presents DRs’ levels, commissions received from the platform, and corresponding points required for each level on a Chinese food delivery platform. Generally, points are credited to their virtual account each time a DR completes a delivery. Additional points can be earned through positive customer evaluations. Mirroring the reward system, there are also two categories of penalties. For instance, rejecting an order results in a fine, while receiving negative reviews or delivery orders late leads to point deductions.

**Table 1:** Delivery riders (DRs’) levels, points, and commissions.

DRs’ levels	Points	Commissions^①^
Bronze	0	None
Silver	500	0.1/order
Gold	1,500	0.2/order
Platinum	3,000	0.3/order
Diamond	5,000	0.4/order
Emperor	10,000	0.5/order

^①^The currency unit is Renminbi (RMB).

This game-like, incentive-driven work system has led many DRs to become deeply engaged, sometimes to the point of overwork, unintentionally extending their working hours. Data shows that in 2018, the average daily working time of DRs in Beijing exceeded 10 h, while in Chengdu, average working hours ranged from 9 to 10 h per day in 2021 (15). As a result, the so-called flexibility associated with gig work may, in practice, constitute a form of “false freedom.” These workers are often described as being “stuck in algorithms.”

### 2.2 Literature review

The well-established JD-R model is commonly employed to examine the effects of work-related pressures on DRs (13, 16). According to the JD-R model, all job characteristics can be classified into two categories: job demands and job resources. Job demands require individuals to exert sustained effort, often resulting in negative outcomes, whereas job resources can help mitigate these effects (17). The model suggests two primary pathways linking job characteristics to employee outcomes: (i) the “health impairment” or negative path assumption and (ii) the “buffer” assumption (17–19).

First, the “negative path” refers to the adverse health effects caused by excessive job demands, often resulting in burnout (20, 21). Several earlier studies on DRs support this assumption. For example, Zheng et al. (22) found that high workloads were significantly associated with traffic crashes. More recent studies have reinforced this view (23). For example, Chen (16) demonstrated that job overload and time pressure were positively associated with job stress among DRs, while self-efficacy helped reduce this impact. Drawing from the Conservation of Resources (COR) theory, Quy Nguyen-Phuoc et al. (24) found that a DR’s perceived control over their work environment played a significant role in the relationship between job demands and burnout. This led to the extension of the JD-R model to incorporate personal demands and resources.

Second, the “buffer” assumption proposes that job resources can attenuate the negative impact of job demands on employee well-being. Prior research has shown that when employees receive social support or timely feedback, job overload is less likely to lead to burnout (21). This buffering effect has also been observed among DRs. Studies using DRs as research subjects confirmed that job resources significantly moderated the relationship between job demands and job burnout (24).

Previous studies have established a link between job demands, job resources, and job outcomes among DRs, generally validating the JD-R model. However, most existing studies have focused on occurrence of injuries such as risky driving behavior or distraction. It remains unclear whether the JD-R model can also explain variations in physical health outcomes. Some recent studies have sought to extend the JD-R model to explore health-related issues targeting formal employed workers. For instance, Lehmann et al. (25) examined workers with chronic health conditions and discovered that job resources decrease the likelihood of illness, while job demands contribute to job burnout and functional limitations. Additionally, some researchers have utilized multiple health indicators, encompassing subjective health (26), fatigue (27), psychological health, and disease risks (28). However, whether this model is also applicable when explore gig workers’ health issues remain unclear. This study aims to address this gap by extending the JD-R model to explore the association between workloads and health status among DRs, thus contributing to the theoretical development of the model.

Specifically, the present study first tests the “negative path” assumption by investigating the correlation between workload and health outcomes among DRs. Following the extended JD-R framework (24), it then examines the underlying mechanisms of this association. As exposure to high-risk working environments may lead to physical fatigue and emotional stress, this study proposes that perceived risk serves as a mediator. Two potential pathways are hypothesized: (i) increased risk perception may prompt DRs to take more safety precautions, thereby reducing their likelihood of illness. (ii) Conversely, elevated perceived risk may trigger anxiety, which in turn contributes to physical health problems.

Finally, the study tests the “buffer” assumption by assessing whether organizational protection measures can alleviate the negative impact of high workload on the health of DRs.

**Figure 1:**
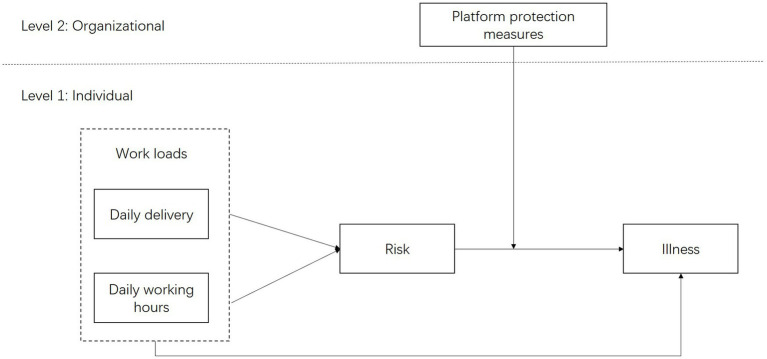
Proposed conceptual framework.

Figure 1 illustrates the conceptual framework of this study. Based on this framework, the following hypotheses are proposed:

*Hypothesis 1*: DRs’ workload is negatively associated with their health status.

*Hypothesis 2*: DRs’ perceived risk mediates the relationship between DRs’ workload and their health status.

*Hypothesis 3*: Platform protection requirements moderate the relationship between DRs’ workload and their health status.

In summary, this study shares some similarities with previous research in that it is also based on the JD-R model to verify the negative impact of workloads on employee health and safety outcomes (13, 16, 17). For example, in consistent with Chen (16) and Zheng et al. (22), this study extends the JD-R model by emphasizing the mediating role of personal risk perceptions. Going beyond previous literature, this study yields some new results: first, existing literature has predominantly focused on occupational burnout (21) or accident risks (22, 29), whereas this research integrates the physiological-psychological association mechanism (H2). Second, this study employs a multidimensional perspective. Previous research has often been based on a single level. This study introduces “platform protection requirements” as a moderating variable (H3), advocating for the necessity of institutional interventions and comprehensively analyzing the influence mechanisms at both the individual and organizational levels.

## 3 Method

### 3.1 Research design

Previous studies on DRs in China have commonly employed the road intercept sampling method due to its efficiency in collecting data over a short period. However, this non-randomized approach can introduce potential bias in the dataset. To address this limitation, the study adopted the respondent-driven sampling (RDS) method to improve the accuracy and representativeness of the sample and to better reflect the overall characteristics of the DR population (30).

To ensure sample quality, RDS analysis tool software was used to conduct an equilibrium sample distribution test on the survey data (31). Following established procedures, the weighted mean absolute discrepancies between the actual ( $P_{s}$ ) and equilibrium sample compositions ( $P_{e}$ ) were used to examine how the actual sample compositions approximated the theoretically computed equilibrium sample compositions ( $\{P_{s}-P_{e}\}<0.02$ ).

The representativeness of the RDS sample was further evaluated by comparing the final RDS sample compositions with asymptotically unbiased estimates of the population composition (t-test for $P_{s}-\bar{P}$ non-significant). The results indicated that the sample used in this survey showed good representativeness.

### 3.2 Data collection

This study employed a structured questionnaire comprising six main sections: (1) socio-demographic information (e.g., age, education), (2) work characteristics (e.g., job type, platform), (3) insurance coverage, (4) occupational injury (e.g., type, number, reason, etc.), and (5) health status (self-rated health, illness, etc.).

A team of 18 trained graduate and undergraduate students recruited 0-round respondents (marked as “seeds”) at the DR’s gathering points. These seeds were selected through one-on-one interviews conducted by trained investigators. The seeds referred to 1^st^-round respondents after finishing their questionnaire; then, 1st-round respondents referred the next round of respondents. The investigators reviewed each respondent to meet the requirements of the RDS. These steps are repeated to form long chains.

To comply with research ethics standards, investigators clearly explained the study’s purpose to all participants. Respondents were informed that participation was voluntary, responses would remain anonymous, and all data would be kept confidential. Before completing the questionnaire, each respondent was required to provide informed consent via a written consent form.

To compensate for their time, each received an incentive of CNY 20 (CNY 20 = USD 3) after their questionnaire was reviewed by the investigator. After the recommended respondents had completed their questionnaires and the completed questionnaires were reviewed, the recommender received an additional reward of CNY 5 (CNY 5 = USD 0.7) (for each successful referral). To control data bias, the investigators reviewed each questionnaire by “daily working hours” and “daily delivery” items and excluded invalid ones.

To ensure the integrity of the referral process and prevent manipulation, such as repeated submissions or referring unrelated individuals, each respondent was permitted to refer a maximum of five other participants. Respondents were assigned a unique identification code and chain number to track referral paths. The final effective sample size for this study was 1,092 participants.

### 3.3 Analytical strategy

The data analysis was conducted in three main steps. First, preliminary model assessments were performed to check for redundancy among variables, including a one-sample t-test, chi-square test, and multicollinearity test.

Second, benchmark regressions were run to examine the relationship between workload and DRs’ health. Given that the dependent variable “illness” is binary, this study used a binary Logit regression model constructed as follows:

(1) $$\text{Illnes}s_{i}^{*}=\alpha +{\text{βworkload}}_{i}+{\mathrm{\gamma Z}}_{i}+\varepsilon_{i}$$

where ${\text{Illness}}_{i}$ is whether rider *I* has ever experienced illness as a result of his work, ${\text{workload}}_{i}$ (main independent variable) is rider *i*’s workload regarding daily delivery (DD) and daily working hours (DWH), $Z_{i}$ is a vector of covariates listed in the section below, and $\varepsilon_{i}$ is the error term.

Third, the study further investigated the mechanisms behind the main correlation. As stated above, this study investigated the mediation role of personal demands between workloads and DRs’ health by using risk perception as a mediator. The study further explored the moderation mechanism by adding organizational-level measures to the main regression. All analyses were performed using Stata version 16.0.

### 3.4 Variables

#### 3.4.1 Illness/health status

The dependent variable in this study is the rider’s experience of work-related illness. This was measured by the question: “What diseases have you suffered or experienced as occupational hazards after working as a rider?” Those who chose any disease are classified as “illness,” and those who chose none are classified as “not illness.”

#### 3.4.2 Workload

The main independent variable, “workload,” was measured using two indicators: DD and DWH. These were derived from the following questions, respectively: “What is your approximate average DD?” and “What is your approximate average DWH?” Both were continuous variables.

#### 3.4.3 Perceived risk

The current study used “perceived risk” as a mediator by asking respondents: “What do you think the level of risk of occupational injury involved in working as an online food DR is?” Riders were able to choose from the following answers: 1 (very low), 2 (low), 3 (normal), 4 (high), or 5 (very high).

#### 3.4.4 Platform (protection) requirements

To understand the platform requirements that may offer riders protection, the respondents were required to answer a series of questions concerning their real working status. For example, “Does the platform have any delivery region restrictions, that is within how many kilometers, or within certain commercial districts?” The Kaiser—Meyer—Olkin (KMO) and Bartlett sphere tests were performed, and the results indicated that these questions were suitable for analysis with KMO = 0.665, *p* < 0.001. We synthesized each item on the scale into one variable by conducting principal component analysis (PCA). Finally, the moderation variable, “platform (protection) requirements,” was established.

#### 3.4.5 Control variables

Based on previous studies on DRs (32, 33), the following control variables were included in this analysis: age, sex, education, marital status, number of underaged children, whether the DR is the main source of income for the family (main supporter), a DR’s type (Zhuansong, Zhongbao), and city. Table 2 lists the assignments for the main variables.

**Table 2:** Assignments for each variable.

Variable	Mean	SD	Assignment
Illness	0.502	0.500	1 = Yes, 0 = No
DD	38.808	16.591	Average daily delivered order number
DWH	10.146	2.669	Average daily working hours
Platform (protection) requirements	0.000	0.382	Based on PCA results
Age	29.875	7.072	
Sex (female)	0.885	0.320	1 = Male, 0 = Female
Marital status (other)	0.533	0.499	1 = Unmarried, 0 = Other
Education (graduate)	2.290	0.523	1 = Primary, 2 = Secondary, 3 = College, 4 = Graduate
Child	1.625	0.827	DRs’ underaged children’s numbers
Main supporter	0.413	0.493	1 = Yes, 0 = No
Job type (part-time)	0.761	0.427	1 = Full-time, 0 = Part-time
Drivers’ type (Zhongbao)	0.411	0.492	1 = Zhuansong, 0 = Zhongbao

## 4 Results

### 4.1 Descriptive statistics

Table 2 presents descriptive statistics for the study sample. Among the 1,092 DRs, the vast majority were males (88.5%). The sample was relatively young, with an average age of 29.875 years (std = 7.072). Most riders were unmarried (53.3%) and employed full-time (76.1%). Additionally, a majority (68.9%) did not hold a college degree.

On average, riders delivered 39 orders per day and worked approximately 10 h daily. Nearly half of the respondents (around 50%) reported experiencing an illness related to their work as DRs.

**Figure 2:**
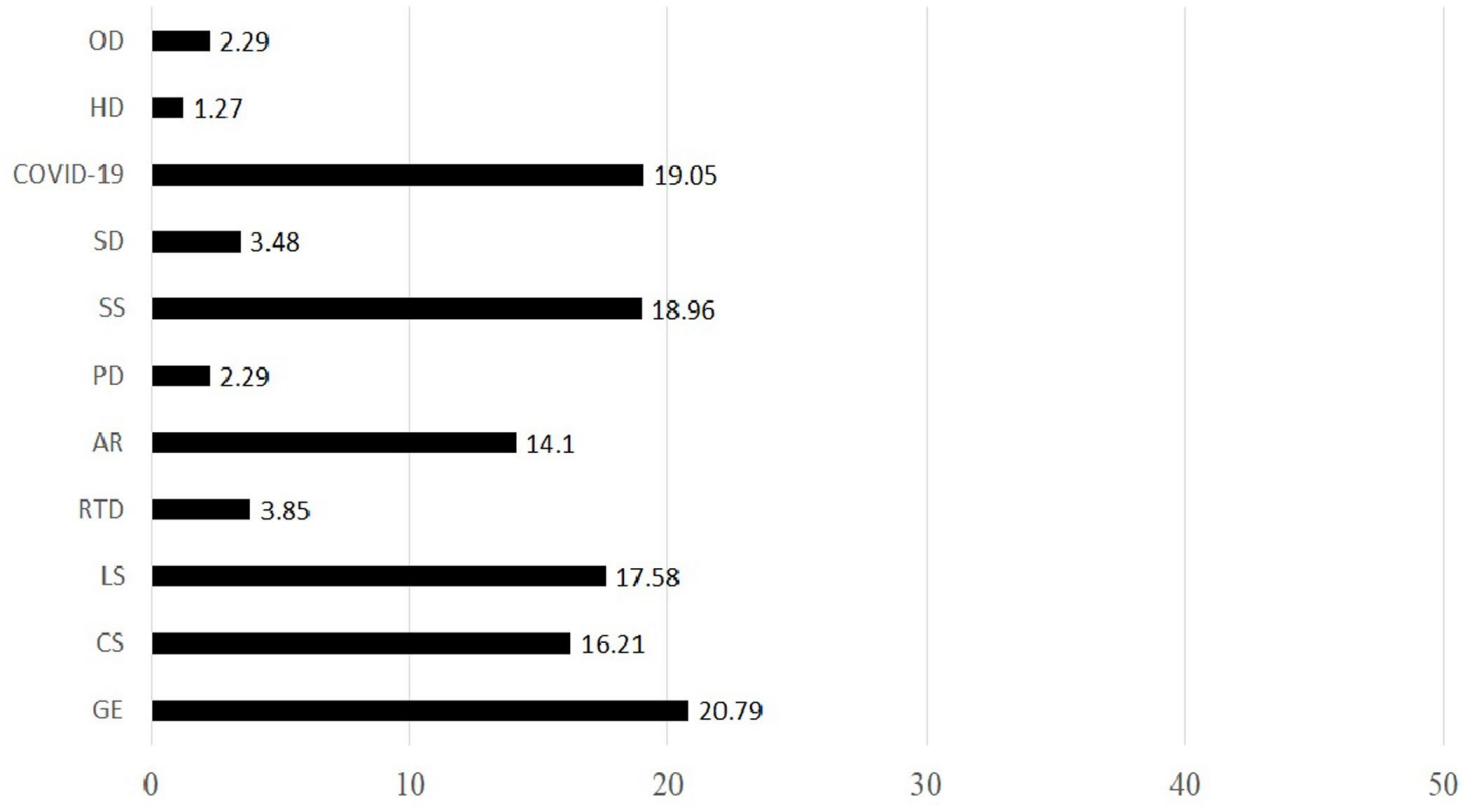
Proportion of job-related disease types. GE, gastro enteropathy; CS, cervical spondylosis; LS, lumbar spondylosis; RTD, respiratory tract diseases; AR, arthritis; PD, prostate disease; SS, sunstroke; SD, skin diseases; HD, heart disease; OD, other diseases.

Figure 2 provides further detail on the types of illnesses reported. Among respondents who experienced work-related illnesses, the most frequently cited conditions were gastroenteropathy (20.79%), COVID-19 (19.05%), and sunstroke (18.96%), followed by lumbar spondylosis (17.58%) and cervical spondylosis (16.21%).

### 4.2 Model assessment

The results of the one-sample t-test and chi-square test indicated that DD, DWH, age, and number of underaged children were significantly correlated with illness, at a significance of *p* < 0.05. Similarly, the chi-square tests for main supporter and marital status were significant (p < 0.05). Additionally, the results of sex (*p* = 0.366) and education (*p* = 0.494) were not significant.

For multicollinearity diagnostics, it was found that when moving the education variable from the regression model, the variance inflation factor (VIF) values of all the variables were lower than 10, and the average VIF value of the model was 1.61, meaning that it passed the test. While when the education variable was included in the regression, the VIF value reached as high as 35, with an average VIF value of the model at 9.44 (this study adopted a strict threshold, considering VIF > 5 as high risk). Common strategies for dealing with high VIF values include: removing variables (specifically, removing the variable with the highest VIF); merging variables (using PCA or factor analysis to transform related variables into new variables, although this approach is not applicable to the “education” variable in our study); and increasing the sample size (which was not feasible in this study). Comprehensively, since sex was a critical demographic variable, it was included in the final regression model while education was moved out.

### 4.3 Benchmark regression between workloads and illness

As shown in Tables 3, 4, there was a significant positive correlation between DD and illness ( β = 0.010, SE = 0.004, p<0.05 ). Regarding the marginal results, with the other variables held constant, the likelihood of suffering from illness increased by 0.2% when DD increased by one unit. DWH is also positive and significantly correlating with illness ( β = 0.051, SE = 0.025, p<0.05 ). When controlling for other variables, the likelihood of suffering from an illness increased by 1.2% for each additional working hour.

**Table 3:** The association between riders’ illness and daily delivery.

Variables	Coefficient	95% CI	dy/dx	95% CI	OR	95% CI
DD	0.010** (0.004)	[0.002, 0.018]	0.002** (0.001)	[0.000, 0.004]	1.010 (0.004)	[1.002,1.018]
Age	0.061*** (0.013)	[0.037, 0.086]	0.014*** (0.003)	[0.009, 0.020]	1.063 (0.013)	[1.039,1.089]
Sex	−0.269 (0.207)	[−0.676, 0.138]	−0.063 (0.048)	[−0.158, 0.032]	0.764 (0.158)	[0.510,1.145]
Marital status	−0.003 (0.221)	[−0.436, 0.429]	−0.001 (0.052)	[−0.102, 0.100]	0.997 (0.215)	[0.653,1.522]
Child	−0.212* (0.122)	[−0.450, 0.027]	−0.05 (0.028)	[−0.105, 0.006]	0.809 (0.096)	[0.641,1.021]
Main supporter	0.217 (0.136)	[−0.049, 0.483]	0.051 (0.032)	[−0.011, 0.113]	1.242 (0.170)	[0.951,1.623]
Riders’ type	Controlled					
City fixed effect	Controlled					
Constant	−1.706*** (0.612)	[−2.905, −0.507]				
Observation	1,084					

^*^Significant at the 10% level; ^**^Significant at the 5% level; ^***^Significant at the 1% level; standard errors display in parentheses; CI, confidence interval; dy/dx, marginal effect; OR, odd ratio.

**Table 4:** The association between riders’ illness and daily working hour.

Variables	Coefficient	95% CI	dy/dx	95% CI	OR	95% CI
DWH	0.051** (0.025)	[0.001, 0.101]	0.012** (0.006)	[0.000, 0.024]	1.052 (0.027)	[1.001,1.106]
Age	0.060*** (0.013)	[0.035, 0.085]	0.014*** (0.003)	[0.009, 0.020]	1.062 (0.013)	[1.037,1.087]
Sex	−0.271 (0.207)	[−0.676, 0.134]	−0.063 (0.048)	[−0.158, 0.031]	0.763 (0.157)	[0.509,1.143]
Marital status	−0.001 (0.222)	[−0.436, 0.433]	−0.000 (0.052)	[−0.102, 0.101]	0.999 (0.216)	[0.654,1.524]
Child	−0.204 (0.123)	[−0.445, 0.036]	−0.048 (0.029)	[−0.104, 0.008]	0.815 (0.097)	[0.646,1.029]
Main supporter	0.204 (0.135)	[−0.061, 0.469]	0.048 (0.032)	[−0.014, 0.110]	1.226 (0.167)	[0.939,1.601]
Riders’ type	Controlled					
City fixed effect	Controlled					
Constant	−1.818*** (0.634)	[−3.061, −0.576]				
Observation	1,085					

*Significant at the 10% level; **Significant at the 5% level; ***Significant at the 1% level; standard errors display in parentheses; CI, confidence interval; dy/dx, marginal effect; OR, odd ratio.

These results indicate that increased workload, both in terms of delivery volume and working hours, is significantly linked to greater health risks among delivery riders. Thus, the findings provide empirical support for Hypothesis 1.

### 4.4 Mediation effect of perceived risk

The bootstrap analysis was used to test the mediation effect of perceived risk in the relationship between workloads and illness, and results showed that the results of both DD and DWH were significant (DD: β=0.001,SE=0.000,p<0.01 ; DWH: β=0.008,SE=0.002,p<0.01 ). Results indicated that a full mediation effect exists between DRs’ workloads, level of risk perception, and illness.

The proportion of mediation effect when using DD and DWH as independent variables were 0.378 and 0.681, respectively. To test the robustness of the results, this study further used Karlson–Holm–Breen (KHB) analysis to test the mediation effect as the illness was treated as a binary dummy variable. As shown in Tables 5A,B, the mediation effects of both DD and DWH were significant when using perceived risk as a mediator. These results confirm that a delivery of a DR’s level of perceived risk mediates the relationship between workload and health. Therefore, Hypothesis 2 is supported.

**Table 5:** Results of mechanism analysis.

Variables	Estimates	SE	95% CI
Panel A: mediation effects for DD			
Results of Bootstrap analysis			
Total effect	0.003**	0.001	
Direct effect	0.002*	0.001	[0.000,0.001]
Indirect effect	0.001***	0	[0.000,0.003]
Results of KHB analysis			
Full effect	0.011***	0.004	[0.002,0.019]
Direct effect	0.007*	0.004	[−0.001,0.016]
Indirect effect	0.003***	0.001	[0.001,0.005]
Panel B: mediation effects for DWH			
Results of Bootstrap analysis			
Total effect	0.012**	0.006	
Direct effect	0.004	0.006	[−0.008,0.015]
Indirect effect	0.008***	0.002	[0.005,0.012]
Results of KHB analysis			
Full effect	0.056**	0.026	[0.004,0.107]
Direct effect	0.019	0.027	[−0.033,0.071]
Indirect effect	0.037***	0.008	[0.022,0.053]
Panel C: moderation effects for DD			
DD	0.009**	0.004	[0.000, 0.017]
Platform requirements	0.503	0.487	[−0.452, 1.458]
DD*platform requirements	−0.004	0.012	[−0.028, 0.019]
Control variables	Controlled		
Observation	1,084		
Panel D: moderation effects for DWH			
DWH	0.027	0.027	[−0.026, 0.080]
Platform requirements	2.350***	0.707	[0.963, 3.736]
DWH*platform requirements	−0.195***	0.067	[−0.327, −0.063]
Control variables	Controlled		
Observation	1,085		

^*^Significant at the 10% level; ^**^Significant at the 5% level; ^***^Significant at the 1% level; CI, confidence interval; dy/dx, marginal effect; SE, standard error.

### 4.5 Moderation effect of work requirements

As shown in Table 5D, extra platform requirements ( β=−0.195 , SE = 0.067) limiting overwork tendency may significantly influence the path between DWH and illness. In the case of DD (Table 5C), the platform requirements did not significantly affect the likelihood of illness. Although not significant, the results for delivery also showed a negative pattern. Hence, both results suggest that a platform limiting riders’ overworking behaviors indirectly aids in alleviating the influence of workloads on illness. Therefore, Hypothesis 3 was partly verified.

### 4.6 Heterogeneity analysis

**Figure 3:**
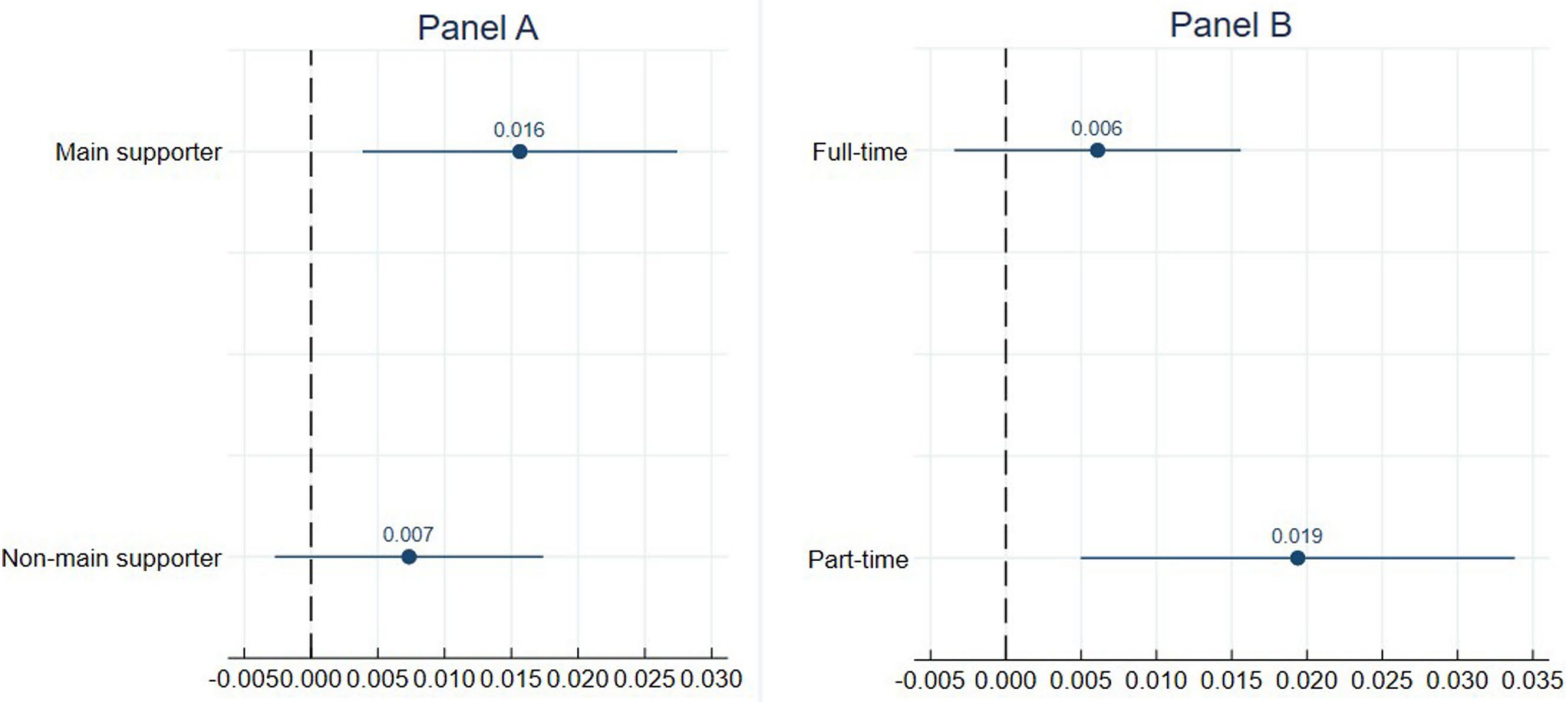
Association between workload and illness among different subgroups. This figure plots point estimates and 95% confidence intervals of DD’s impact on the probability of getting illness between different groups. These coefficients are derived from Logit estimates of Equation 1 after it has been fully interacted with the moderator variables of interest. The moderator variable in Panel **(A)** is whether the rider is his family’s main financial supporter. The moderator variable in Panel **(B)** is riders’ job type—full-time or part-time. The 95% intervals are constructed using robust standard errors.

Figure 3A decomposes the association between DD and illness based on whether the rider is the main financial supporter of the family. The first and second coefficients correspond to riders who are and are not the primary income earners, respectively. The figure reveals that riders, who are the main source of income for their families, are more susceptible to illness due to work overload.

Figure 3B highlights that workload has less of an effect on DRs who are working as full-time riders but leads to higher risks of illness among counter groups.

## 5 Discussion

Occupational injury among DRs has become a global concern, yet limited research has explored the health-related consequences of their working conditions or the factors that influence them. This study examined the association between workloads and illness among DRs in China and investigated the underlying mechanisms to provide evidence-based recommendations for labor protection.

Survey results revealed that gastroenteropathy was the most commonly reported occupational health hazard among DRs. This condition is likely linked to disrupted eating patterns, as riders often work through traditional mealtimes due to high demand during dining hours. COVID-19 was the second most reported illness. When grouped with other respiratory tract diseases, this category surpasses gastrointestinal diseases as the most frequently reported. This finding aligns with a previous study conducted in Ecuador, which documented a high prevalence of SARS-CoV-2 infection among DRs (4).

The results of this study show that workload is positively and significantly correlated with illness, indicating that higher levels of daily deliveries and longer working hours increase the likelihood of health problems among DRs. These findings are consistent with earlier research. For instance, a study in Vietnam demonstrated that job burnout and personal demands directly impact risky riding behavior, with job burnout being the most significant predictor (24). Similarly, research in China has shown that job overload and time pressure positively impact riders’ job stress, increasing the risk of unsafe driving and distraction (16). Possible explanations for this result can be found in relevant studies that lead to dangerous driving behavior. Existing research has shown that work stress can lead to anxiety, meanwhile Traficante et al. (34) and Koppel et al. (35) revealed that some psychological factors can influence driver’s behavior. For example, anxiety may worsen a driver’s safety driving behavior, with the driver’s self-regulation abilities mediated the influence of anxiety on driving lapses.

Beyond this previous research, this study makes a theoretical contribution by extending the JD-R model to explain the occupational health hazard among DRs. The significant correlation between workloads and illness supports the “negative pathway” assumption of the JD-R model that excessive job demands can lead to adverse health outcomes.

The mediation analysis revealed a significant mediation effect between DR’s workload and illness, with risk perception acting as a mediator. This finding suggests that in the relation between DR’s workloads and health, a mechanism exists on an individual level. Specifically, the results support the assumption that as DRs experience greater workload intensity, their perceived level of occupational risk also increases. This heightened perception can lead to psychological reactions such as anxiety and distraction, which may eventually manifest as physical illness.

These findings align with existing psychological research showing that psychological stress is closely associated with negative health outcomes. Stress can affect health directly via autonomic and neuroendocrine pathways and indirectly by influencing health behaviors (36). Daily stressors impact physical health and even lifespan by influencing the autonomic nervous system, endocrine system, and immune system (37). For example, psychological stress has been linked to increased incidence of common colds and heightened risk for chronic conditions such as arthritis, cardiovascular disease, and diabetes (38–40). This study adds to the growing body of evidence supporting the link between psychological stress and health while empirically confirming a mediation effect from workloads to illness through risk perception. These results underscore the importance of addressing mental health concerns among delivery riders in discussions of occupational health and safety.

Notably, organizational-level mechanisms that may affect the association between DR’s workloads and health also exist. Specifically, platform interventions aimed at regulating riders’ behavior, particularly those that limit overwork, were found to buffer the negative health effects of high workload. For example, interviews reveal that some platforms in China implement delivery region limits or require riders to work in shifts, both of which help reduce physical strain. Our quantitative analysis showed that such organizational measures significantly reduce the likelihood of illness among DRs.

These findings highlight the critical role that platforms play in shaping working conditions and promoting rider well-being. Although only minor measures were taken, and even the platform’s original intention was to limit the workload of riders rather than protect them, the ultimate result will still benefit riders. This result provides empirical evidence for prior studies that, when mapped across the gig work system, the most common hazards were at the company level (41). Furthermore, when companies and colleagues provide support, riders are more likely to engage in preventive health behaviors (8).

Therefore, injury prevention efforts should not solely rely on encouraging riders to increase their personal safety awareness. Instead, platforms must take active responsibility by designing structural protections that reduce work-related risks and promote a safer, healthier work environment for DRs.

The subgroup analysis revealed a more complex relationship between family situation, job type, workload, and illness. The correlation between DD and illness was more statistically significant among riders who were their families’ main financial supporters. One possible explanation is that these individuals have a stronger incentive to maintain employment and are more motivated to increase their delivery volume to maximize earnings. This greater workload intensity may expose them to higher stress levels and physical strain, ultimately increasing their risk of illness (8).

Similarly, DD had a more substantial effect on part-time riders, suggesting that employment instability and a lack of organizational support may heighten health vulnerability in this group. These findings align with previous research by Koranyi et al. (42) and Zhan et al. (33), who found that employment instability and income dependence are positively associated with occupational injuries. This study adds to the existing literature by highlighting that among precariously employed gig workers, part-time riders are more unstable and even more vulnerable.

Despite its contributions, this study had three main limitations. First, illness was used as the dependent variable to draw conclusions about health risks and potential prevention strategies. However, the applicability of these findings to injury prevention remains uncertain and warrants further investigation. Second, the sample was divided into subgroups to conduct a heterogeneity analysis. However, the smaller sample sizes within subgroups reduced statistical power, limiting the generalizability of those specific findings. Therefore, results from subgroup analyses should be interpreted with caution. Future research should further explore these subgroup dynamics with larger samples and consider extending the focus to injury-related outcomes. Third, the results of this study may not be precise enough because of the use of 0–1 self-report illness. Therefore, we suggest that future research should use a more fully designed scale, reflecting more objective health status, and refine the options to further explore the findings of this study.

## 6 Conclusion

The rising health risks faced by DRs have drawn increasing attention from both policymakers and researchers. The findings of this study reveal that the heavy workloads experienced by DRs in China are partly a result of platform-driven work process designs. In particular, the game-like point upgrade system, originally developed to appeal to younger workers, appears to have inadvertently contributed to overwork and behavioral addiction.

Given the demonstrated link between high workloads and increased illness risk, platforms must take responsibility for mitigating health hazards. One crucial step is to improve algorithmic design, making the workflow more balanced and health-conscious.

Additionally, the study highlights that risk perception and susceptibility to overwork vary across individuals, suggesting that targeted interventions should be developed for especially vulnerable subgroups.

Beyond the direct health benefits of managing workload, organizational-level protective measures can buffer the negative impact of work demands. This reinforces the importance of platform accountability in ensuring occupational health and safety. Ultimately, protecting the well-being of riders is not only an ethical imperative but also a foundational requirement for the sustainable development of labor on these platforms.

In regarding of recommendations for future research, since this study only investigates online food DRs; further research is necessary to determine whether the results are also applicable to other gig economy participants. And future research may need to embody more objective health indicators to further investigate gig workers’ health issue. Finally, further study using longitudinal health data may reveal more health issues regarding gig workers.

## Data Availability

The datasets presented in this article are not readily available because the 0-round respondents (marked as “seeds”) were interviewed one on one, and involve personal information that owing to the privacy and confidential agreements, we regretfully cannot furnish the raw data. Requests to access the datasets should be directed to corresponding author Ailin Mao (maoailin2006@126.com) upon reasonable request.
